# (1*R*,2*R*)-*N*,*N*′-Diisobutyl-*N*,*N*′-dimethyl­cyclo­hexane-1,2-diamine

**DOI:** 10.1107/S160053680901109X

**Published:** 2009-04-02

**Authors:** Prisca K. Eckert, Viktoria H. Gessner, Carsten Strohmann

**Affiliations:** a Anorganische Chemie, Technische Universität Dortmund, Otto-Hahn-Strasse 6, 44227 Dortmund, Germany

## Abstract

The title compound, C_16_H_34_N_2_, is a chiral diamine with fixed *R* configuration at both stereogenic carbon centres of the cyclo­hexane backbone. Due to their different substituents, the two N atoms also become stereogenic. In the crystal structure, the configuration at one of the two nitro­gen centres is fixed, with the free electron pair pointing inward and the isobutyl group in a *trans* position towards the cyclo­hexane backbone resulting in an *R* configuration. The isobutyl group at the second N atom, however, is disordered with 75% *S* configuration and 25% *R* configuration. In both cases, the isobutyl group is arranged in a *trans* position towards the cyclo­hexane backbone.

## Related literature

The synthesis of the title compound is described by Kizirian *et al.* (2003 ▶). For the crystal structure of the related mol­ecule (1*R*,2*R*)-*N*,*N′*-dimethyl­cyclo­hexane-1,2-diamine, see Strohmann *et al.* (2008*b* ▶). Crystal structures of (1*R*,2*R*)-*N*,*N′*-tetra­methyl­cyclo­hexane-1,2-diamine coordinated to lithium organyls are described by Strohmann & Gessner (2007*a* ▶) and Strohmann & Gessner (2008 ▶). Other related diamines coordinated to lithium organyls are specified by Strohmann & Gessner (2007*b* ▶) and Strohmann *et al.* (2008*a* ▶). The use of chiral nitrogen ligands to enhance the stereoselectivity of deprotonation or addition reactions is discussed by Kizirian (2008 ▶) and Stead *et al.* (2008 ▶).
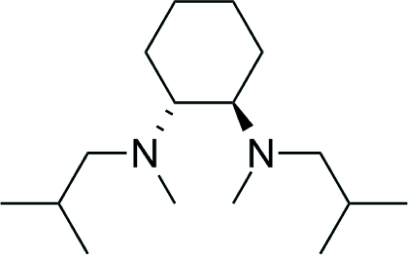


## Experimental

### 

#### Crystal data

C_16_H_34_N_2_
*M* *_r_* = 254.45
Orthorhombic, 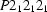

*a* = 10.4693 (15) Å
*b* = 10.8013 (16) Å
*c* = 15.077 (2) Å
*V* = 1705.0 (4) Å^3^
*Z* = 4
Mo *K*α radiation
μ = 0.06 mm^−1^
*T* = 173 K
0.40 × 0.40 × 0.20 mm

#### Data collection

Bruker SMART APEX CCD diffractometer
Absorption correction: multi-scan (*SADABS*; Bruker, 1999 ▶) *T*_min_ = 0.977, *T*_max_ = 0.989
20171 measured reflections
3004 independent reflections
2730 reflections with *I* > 2σ(*I*)
*R*_int_ = 0.051

#### Refinement

*R*[*F* ^2^ > 2σ(*F* ^2^)] = 0.056
*wR*(*F* ^2^) = 0.149
*S* = 1.06
3004 reflections
202 parameters
6 restraints
H atoms treated by a mixture of independent and constrained refinement
Δρ_max_ = 0.20 e Å^−3^
Δρ_min_ = −0.13 e Å^−3^
Absolute structure: not determined in the present model. Absolute configuration: known from starting material

### 

Data collection: *SMART* (Bruker, 2001 ▶); cell refinement: *SAINT-Plus* (Bruker, 1999 ▶); data reduction: *SAINT-Plus*; program(s) used to solve structure: *SHELXS97* (Sheldrick, 2008 ▶); program(s) used to refine structure: *SHELXL97* (Sheldrick, 2008 ▶); molecular graphics: *ORTEP-3* (Farrugia, 1997 ▶); software used to prepare material for publication: *WinGX* (Farrugia, 1999 ▶).

## Supplementary Material

Crystal structure: contains datablocks I, global. DOI: 10.1107/S160053680901109X/fi2074sup1.cif

Structure factors: contains datablocks I. DOI: 10.1107/S160053680901109X/fi2074Isup2.hkl

Additional supplementary materials:  crystallographic information; 3D view; checkCIF report

## Figures and Tables

**Fig. 1.**
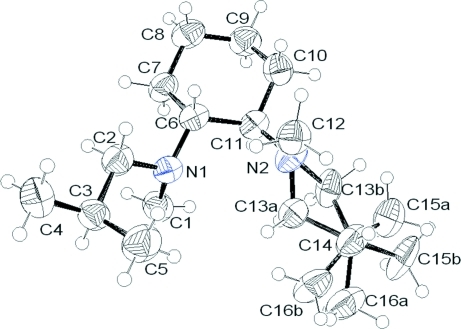
The molecular structure of the title compound with thermal ellipsoids drawn at the 50% probability level.

## supplementary crystallographic information

### Comment

In preparative chemistry, chiral nitrogen ligands such as (1*R*,2*R*)*-N,N,N',N'-*tetramethylcyclohexane-1,2-diamine [(*R*,*R*)-TMCDA] and its derivatives are often used to enhance the stereoselectivity of deprotonation or addition reactions by coordinating to organolithium reagents (Kizirian, 2008). In the case of the cyclohexanediamine ligands, derivatives with three different substituents at the nitrogen centres revealed to be more efficient than their symmetric analogues (Kizirian *et al., *2003; Stead *et al.*, 2008).

The title compound represents the crystal structure of such an uncoordinated (*R*,*R*)-TMCDA derivative (for related crystal structures, see: Strohmann & Gessner, 2007*a*,*b*; Strohmann & Gessner, 2008; Strohmann *et al.**et al.*, 2008*b*; Strohmann *et al.*, 2008*a*). (1*R*,2*R*)-*N*,*N'*-diisobutyl-*N*, *N'*-dimethylcyclohexane-1,2-diamine (1) (Fig. 1), crystallizes at -78 °C in the orthorhombic crystal system, space group *P*2_1_2_1_2_1_. The asymmetric unit contains one molecule of the chiral diamine. The configuration at one of the two nitrogen centres is fixed with the free electron pair pointing inward and the isobutyl group arranged in a *trans*-position towards the cyclohexane backbone. The second nitrogen centre, however, can be described by a model that has the free electron pair pointing outwards in 75% of all molecules and inwards in the others.

### Experimental

The title compound (100 mg, 0.39 mmol) was diluted in *n*-pentane (2 ml). After cooling to -78 °C, clear crystals suitable for single-crystal *x*-ray studies were obtained. For synthesis of the title compound, see Kizirian *et al.* (2003).

### Refinement

H atoms were refined using a riding model in their ideal geometric positions with *U*_iso_(H) = 1.5*U*_eq_(C) for methyl H atoms and *U*_iso_(H) = 1.2*U*_eq_(C) for all other H atoms. H14 was located from the Fourier map and refined and its coordinates were refined freely yielding a C—H distance of 1.03 (3) Å. The Friedel pairs were not merged and the Flack parameter (Flack, 1983) yielded an indeterminate value with large uncertainty (1(4)). The following distances were restrained using DFIX: C13b—N2 and N2—C13a at 1.43 Å, C14—C16b, C14—16a, C14—C15a and C14—C15b at 1.52 Å. For the description of the disorder, a splitting model was used which had the free electron pair at the nitrogen centre pointing outwards in 75% of all molecules and inwards in the others. Absolute structure: not determined in the present model. Absolute configuration: known from starting material.

### Crystal data

**Table:**

C_16_H_34_N_2_	*F*(000) = 576
*M**_r_* = 254.45	*D*_x_ = 0.991 Mg m^−^^3^
Orthorhombic, *P*2_1_2_1_2_1_	Mo *K*α radiation, λ = 0.71073 Å
Hall symbol: P2ac 2ab	Cell parameters from 999 reflections
*a* = 10.4693 (15) Å	θ = 2.3–25°
*b* = 10.8013 (16) Å	µ = 0.06 mm^−^^1^
*c* = 15.077 (2) Å	*T* = 173 K
*V* = 1705.0 (4) Å^3^	Plates, colourless
*Z* = 4	0.40 × 0.40 × 0.20 mm

### Data collection

**Table:**

Bruker SMART APEX CCD diffractometer	3004 independent reflections
Radiation source: fine-focus sealed tube	2730 reflections with *I* > 2σ(*I*)
graphite	*R*_int_ = 0.051
ω scans	θ_max_ = 25.0°, θ_min_ = 2.3°
Absorption correction: multi-scan (*SADABS*; Bruker, 1999)	*h* = −12→12
*T*_min_ = 0.977, *T*_max_ = 0.989	*k* = −12→12
20171 measured reflections	*l* = −17→17

### Refinement

**Table:**

Refinement on *F*^2^	Primary atom site location: structure-invariant direct methods
Least-squares matrix: full	Secondary atom site location: difference Fourier map
*R*[*F*^2^ > 2σ(*F*^2^)] = 0.056	Hydrogen site location: inferred from neighbouring sites
*wR*(*F*^2^) = 0.149	H atoms treated by a mixture of independent and constrained refinement
*S* = 1.06	*w* = 1/[σ^2^(*F*_o_^2^) + (0.0787*P*)^2^ + 0.3166*P*] where *P* = (*F*_o_^2^ + 2*F*_c_^2^)/3
3004 reflections	(Δ/σ)_max_ < 0.001
202 parameters	Δρ_max_ = 0.20 e Å^−^^3^
6 restraints	Δρ_min_ = −0.13 e Å^−^^3^

### Special details

**Table:**

Geometry. All e.s.d.s (except the e.s.d. in the dihedral angle between two l.s. planes) are estimated using the full covariance matrix. The cell e.s.d.s are taken into account individually in the estimation of e.s.d.s in distances, angles and torsion angles; correlations between e.s.d.s in cell parameters are only used when they are defined by crystal symmetry. An approximate (isotropic) treatment of cell e.s.d.s is used for estimating e.s.d.s involving l.s. planes.
Refinement. Refinement of *F*^2^ against ALL reflections. The weighted *R*-factor *wR* and goodness of fit *S* are based on *F*^2^, conventional *R*-factors *R* are based on *F*, with *F* set to zero for negative *F*^2^. The threshold expression of *F*^2^ > σ(*F*^2^) is used only for calculating *R*-factors(gt) *etc*. and is not relevant to the choice of reflections for refinement. *R*-factors based on *F*^2^ are statistically about twice as large as those based on *F*, and *R*- factors based on ALL data will be even larger.

### Fractional atomic coordinates and isotropic or equivalent isotropic displacement parameters (Å^2^)

**Table:**

	*x*	*y*	*z*	*U*_iso_*/*U*_eq_	Occ. (<1)
C1	0.7243 (2)	0.2944 (2)	0.79640 (18)	0.0615 (6)	
H1A	0.6695	0.3257	0.7487	0.092*	
H1B	0.8141	0.3069	0.7804	0.092*	
H1C	0.7055	0.3390	0.8514	0.092*	
C2	0.5745 (2)	0.1385 (2)	0.84275 (17)	0.0589 (6)	
H2A	0.5630	0.1850	0.8988	0.071*	
H2B	0.5683	0.0493	0.8570	0.071*	
C3	0.4646 (2)	0.1725 (2)	0.77945 (15)	0.0546 (6)	
H3	0.4648	0.2643	0.7704	0.065*	
C4	0.3389 (2)	0.1358 (3)	0.8223 (2)	0.0727 (8)	
H4A	0.2682	0.1572	0.7825	0.109*	
H4B	0.3288	0.1802	0.8785	0.109*	
H4C	0.3385	0.0464	0.8334	0.109*	
C5	0.4814 (3)	0.1104 (3)	0.69055 (19)	0.0778 (8)	
H5A	0.4885	0.0207	0.6990	0.117*	
H5B	0.5592	0.1415	0.6621	0.117*	
H5C	0.4075	0.1286	0.6529	0.117*	
C6	0.7989 (2)	0.0998 (2)	0.86174 (14)	0.0489 (5)	
H6	0.7655	0.0145	0.8733	0.059*	
C7	0.8261 (2)	0.1571 (2)	0.95317 (15)	0.0559 (6)	
H7A	0.8633	0.2406	0.9452	0.067*	
H7B	0.7449	0.1662	0.9861	0.067*	
C8	0.9177 (2)	0.0778 (3)	1.00701 (16)	0.0629 (6)	
H8A	0.9356	0.1187	1.0644	0.075*	
H8B	0.8778	−0.0035	1.0194	0.075*	
C9	1.0415 (3)	0.0585 (3)	0.95720 (17)	0.0649 (7)	
H9A	1.0974	0.0020	0.9913	0.078*	
H9B	1.0862	0.1388	0.9509	0.078*	
C10	1.0166 (2)	0.0039 (2)	0.86573 (17)	0.0597 (6)	
H10A	0.9798	−0.0800	0.8726	0.072*	
H10B	1.0989	−0.0045	0.8339	0.072*	
C11	0.9259 (2)	0.0829 (2)	0.81027 (15)	0.0498 (5)	
H11	0.9656	0.1667	0.8048	0.060*	
C12	0.8408 (3)	−0.0772 (2)	0.71239 (16)	0.0619 (6)	
H12A	0.7509	−0.0585	0.7245	0.093*	
H12B	0.8714	−0.1392	0.7548	0.093*	
H12C	0.8492	−0.1097	0.6520	0.093*	
C14	0.9642 (3)	0.0817 (2)	0.56237 (16)	0.0668 (7)	
H14	0.931 (3)	−0.003 (3)	0.5418 (19)	0.075 (8)*	
C13A	0.8956 (3)	0.1169 (3)	0.64982 (18)	0.0542 (7)	0.75
H13C	0.9256	0.1998	0.6686	0.065*	0.75
H13D	0.8028	0.1231	0.6380	0.065*	0.75
C15A	1.1073 (3)	0.0796 (6)	0.5758 (4)	0.1065 (18)	0.75
H15D	1.1369	0.1625	0.5924	0.160*	0.75
H15E	1.1492	0.0541	0.5206	0.160*	0.75
H15F	1.1287	0.0208	0.6230	0.160*	0.75
C16A	0.9211 (5)	0.1727 (3)	0.4920 (3)	0.0825 (12)	0.75
H16D	0.8284	0.1663	0.4842	0.124*	0.75
H16E	0.9637	0.1535	0.4357	0.124*	0.75
H16F	0.9432	0.2570	0.5104	0.124*	0.75
C13B	1.0029 (8)	0.0830 (10)	0.6582 (5)	0.062 (3)	0.25
H13A	1.0842	0.0369	0.6637	0.074*	0.25
H13B	1.0204	0.1699	0.6751	0.074*	0.25
C15B	1.0898 (9)	0.1100 (18)	0.5153 (9)	0.102 (5)	0.25
H15A	1.0741	0.1202	0.4517	0.153*	0.25
H15B	1.1497	0.0416	0.5247	0.153*	0.25
H15C	1.1263	0.1865	0.5394	0.153*	0.25
C16B	0.8646 (9)	0.1787 (9)	0.5399 (8)	0.079 (4)	0.25
H16A	0.9004	0.2614	0.5497	0.118*	0.25
H16B	0.7896	0.1673	0.5779	0.118*	0.25
H16C	0.8394	0.1701	0.4776	0.118*	0.25
N1	0.70037 (17)	0.16326 (17)	0.80918 (12)	0.0488 (4)	
N2	0.9148 (2)	0.03281 (19)	0.72094 (12)	0.0610 (6)	

### Atomic displacement parameters (Å^2^)

**Table:**

	*U*^11^	*U*^22^	*U*^33^	*U*^12^	*U*^13^	*U*^23^
C1	0.0625 (14)	0.0513 (13)	0.0708 (16)	0.0042 (11)	0.0025 (13)	0.0042 (12)
C2	0.0647 (14)	0.0613 (14)	0.0507 (13)	−0.0004 (11)	0.0043 (11)	−0.0009 (11)
C3	0.0584 (13)	0.0525 (12)	0.0528 (13)	0.0063 (11)	−0.0044 (11)	−0.0018 (11)
C4	0.0608 (14)	0.0777 (17)	0.0795 (19)	0.0068 (13)	0.0026 (14)	−0.0061 (15)
C5	0.0804 (18)	0.096 (2)	0.0568 (15)	−0.0007 (16)	−0.0078 (15)	−0.0057 (15)
C6	0.0569 (12)	0.0472 (12)	0.0425 (11)	−0.0058 (10)	0.0003 (10)	0.0022 (9)
C7	0.0600 (13)	0.0661 (14)	0.0417 (12)	−0.0017 (12)	0.0012 (10)	−0.0021 (11)
C8	0.0668 (15)	0.0772 (16)	0.0447 (13)	−0.0073 (14)	−0.0073 (12)	0.0019 (12)
C9	0.0615 (14)	0.0754 (17)	0.0577 (14)	−0.0009 (13)	−0.0100 (12)	0.0024 (13)
C10	0.0534 (13)	0.0625 (15)	0.0633 (15)	0.0003 (11)	−0.0009 (12)	−0.0041 (12)
C11	0.0545 (12)	0.0490 (11)	0.0458 (12)	−0.0074 (10)	0.0008 (10)	−0.0032 (10)
C12	0.0734 (16)	0.0575 (14)	0.0548 (14)	−0.0028 (12)	−0.0042 (12)	−0.0110 (12)
C14	0.093 (2)	0.0611 (15)	0.0467 (13)	−0.0051 (14)	0.0091 (13)	−0.0048 (12)
C13A	0.0574 (18)	0.0569 (17)	0.0483 (18)	−0.0011 (14)	0.0007 (14)	−0.0032 (14)
C15A	0.093 (4)	0.144 (5)	0.083 (3)	0.033 (3)	0.031 (3)	0.022 (4)
C16A	0.125 (4)	0.077 (2)	0.045 (2)	−0.008 (3)	0.001 (2)	−0.0032 (19)
C13B	0.040 (5)	0.080 (7)	0.065 (6)	−0.012 (4)	0.009 (4)	−0.019 (5)
C15B	0.074 (8)	0.159 (16)	0.072 (9)	−0.006 (9)	−0.008 (7)	−0.035 (10)
C16B	0.081 (8)	0.112 (10)	0.044 (6)	−0.024 (7)	−0.002 (6)	0.001 (7)
N1	0.0529 (10)	0.0485 (10)	0.0449 (10)	−0.0022 (8)	−0.0001 (8)	0.0034 (8)
N2	0.0701 (13)	0.0672 (12)	0.0457 (11)	−0.0163 (10)	0.0073 (10)	−0.0078 (9)

### Geometric parameters (Å, °)

**Table:**

C1—N1	1.451 (3)	C10—H10B	0.99
C1—H1A	0.98	C11—N2	1.456 (3)
C1—H1B	0.98	C11—H11	1
C1—H1C	0.98	C12—N2	1.425 (3)
C2—N1	1.437 (3)	C12—H12A	0.98
C2—C3	1.539 (3)	C12—H12B	0.98
C2—H2A	0.99	C12—H12C	0.98
C2—H2B	0.99	C14—C13B	1.500 (9)
C3—C5	1.509 (4)	C14—C15A	1.512 (3)
C3—C4	1.519 (3)	C14—C16A	1.516 (3)
C3—H3	1	C14—C16B	1.517 (3)
C4—H4A	0.98	C14—C15B	1.525 (3)
C4—H4B	0.98	C14—C13A	1.549 (4)
C4—H4C	0.98	C14—H14	1.03 (3)
C5—H5A	0.98	C13A—N2	1.4196 (18)
C5—H5B	0.98	C13A—H13C	0.99
C5—H5C	0.98	C13A—H13D	0.99
C6—N1	1.470 (3)	C15A—H15D	0.98
C6—C7	1.538 (3)	C15A—H15E	0.98
C6—C11	1.550 (3)	C15A—H15F	0.98
C6—H6	1	C16A—H16D	0.98
C7—C8	1.521 (3)	C16A—H16E	0.98
C7—H7A	0.99	C16A—H16F	0.98
C7—H7B	0.99	C13B—N2	1.428 (2)
C8—C9	1.512 (4)	C13B—H13A	0.99
C8—H8A	0.99	C13B—H13B	0.99
C8—H8B	0.99	C15B—H15A	0.98
C9—C10	1.522 (4)	C15B—H15B	0.98
C9—H9A	0.99	C15B—H15C	0.98
C9—H9B	0.99	C16B—H16A	0.98
C10—C11	1.527 (3)	C16B—H16B	0.98
C10—H10A	0.99	C16B—H16C	0.98
			
N1—C1—H1A	109.5	C10—C11—H11	107
N1—C1—H1B	109.5	C6—C11—H11	107
H1A—C1—H1B	109.5	N2—C12—H12A	109.5
N1—C1—H1C	109.5	N2—C12—H12B	109.5
H1A—C1—H1C	109.5	H12A—C12—H12B	109.5
H1B—C1—H1C	109.5	N2—C12—H12C	109.5
N1—C2—C3	115.0 (2)	H12A—C12—H12C	109.5
N1—C2—H2A	108.5	H12B—C12—H12C	109.5
C3—C2—H2A	108.5	C15A—C14—C16A	113.5 (4)
N1—C2—H2B	108.5	C13B—C14—C16B	113.2 (7)
C3—C2—H2B	108.5	C13B—C14—C15B	102.3 (6)
H2A—C2—H2B	107.5	C16B—C14—C15B	110.5 (9)
C5—C3—C4	111.3 (2)	C15A—C14—C13A	110.4 (3)
C5—C3—C2	110.9 (2)	C16A—C14—C13A	107.4 (3)
C4—C3—C2	108.8 (2)	C13B—C14—H14	113.1 (16)
C5—C3—H3	108.6	C15A—C14—H14	111.5 (17)
C4—C3—H3	108.6	C16A—C14—H14	105.3 (16)
C2—C3—H3	108.6	C16B—C14—H14	108.2 (17)
C3—C4—H4A	109.5	C15B—C14—H14	109.5 (18)
C3—C4—H4B	109.5	C13A—C14—H14	108.5 (17)
H4A—C4—H4B	109.5	N2—C13A—C14	114.8 (2)
C3—C4—H4C	109.5	N2—C13A—H13C	108.6
H4A—C4—H4C	109.5	C14—C13A—H13C	108.6
H4B—C4—H4C	109.5	N2—C13A—H13D	108.6
C3—C5—H5A	109.5	C14—C13A—H13D	108.6
C3—C5—H5B	109.5	H13C—C13A—H13D	107.5
H5A—C5—H5B	109.5	C14—C15A—H15D	109.5
C3—C5—H5C	109.5	C14—C15A—H15E	109.5
H5A—C5—H5C	109.5	H15D—C15A—H15E	109.5
H5B—C5—H5C	109.5	C14—C15A—H15F	109.5
N1—C6—C7	115.18 (19)	H15D—C15A—H15F	109.5
N1—C6—C11	112.73 (17)	H15E—C15A—H15F	109.5
C7—C6—C11	109.68 (18)	C14—C16A—H16D	109.5
N1—C6—H6	106.2	C14—C16A—H16E	109.5
C7—C6—H6	106.2	H16D—C16A—H16E	109.5
C11—C6—H6	106.2	C14—C16A—H16F	109.5
C8—C7—C6	111.6 (2)	H16D—C16A—H16F	109.5
C8—C7—H7A	109.3	H16E—C16A—H16F	109.5
C6—C7—H7A	109.3	N2—C13B—C14	117.4 (6)
C8—C7—H7B	109.3	N2—C13B—H13A	108
C6—C7—H7B	109.3	C14—C13B—H13A	108
H7A—C7—H7B	108	N2—C13B—H13B	108
C9—C8—C7	110.6 (2)	C14—C13B—H13B	108
C9—C8—H8A	109.5	H13A—C13B—H13B	107.2
C7—C8—H8A	109.5	C14—C15B—H15A	109.5
C9—C8—H8B	109.5	C14—C15B—H15B	109.5
C7—C8—H8B	109.5	H15A—C15B—H15B	109.5
H8A—C8—H8B	108.1	C14—C15B—H15C	109.5
C8—C9—C10	110.9 (2)	H15A—C15B—H15C	109.5
C8—C9—H9A	109.5	H15B—C15B—H15C	109.5
C10—C9—H9A	109.5	C14—C16B—H16A	109.5
C8—C9—H9B	109.5	C14—C16B—H16B	109.5
C10—C9—H9B	109.5	H16A—C16B—H16B	109.5
H9A—C9—H9B	108	C14—C16B—H16C	109.5
C9—C10—C11	112.7 (2)	H16A—C16B—H16C	109.5
C9—C10—H10A	109.1	H16B—C16B—H16C	109.5
C11—C10—H10A	109.1	C2—N1—C1	112.73 (19)
C9—C10—H10B	109.1	C2—N1—C6	111.51 (17)
C11—C10—H10B	109.1	C1—N1—C6	113.93 (18)
H10A—C10—H10B	107.8	C13A—N2—C12	112.9 (2)
N2—C11—C10	110.38 (19)	C12—N2—C13B	127.4 (5)
N2—C11—C6	116.00 (19)	C13A—N2—C11	118.2 (2)
C10—C11—C6	108.98 (18)	C12—N2—C11	115.92 (18)
N2—C11—H11	107	C13B—N2—C11	114.9 (4)
